# Supplementary material for: Surface engineering of inorganic solid-state electrolytes via interlayers strategy for developing long-cycling quasi-all-solid-state lithium batteries
Source: Nat Commun. 2023 Feb 11;14:782. doi: 10.1038/s41467-023-36401-7 (PMC9922298; doi:10.1038/s41467-023-36401-7)
Supplement: Supplementary file 1 — Supplementary fiqure and table [file 41467_2023_36401_MOESM1_ESM.pdf]

Supplementary Information

Surface engineering of inorganic solid-state electrolytes via interlayers strategy for developing long-cycling quasi-all-solid-state lithium batteries

Ju-Sik Kim[1†], Gabin Yoon[1†], Sewon Kim[1], Shoichi Sugata[2], Nobuyoshi Yashiro[2], Shinya Suzuki[2], Myung-Jin Lee[1], Ryounghee Kim[1], Michael Badding[3], Zhen Song[3], JaeMyung Chang[3], Dongmin Im[1*]*

1 Battery Material Lab., Samsung Advanced Institute of Technology, 130, Samsung-ro, Yeongtong-gu, Suwon-si, Gyeonggi-do, Republic of Korea, 443-803
2 Samsung R&D Institute Japan, Samsung Electronics, 2-1-11, Semba Nishi, Minoh, Osaka, Japan
3 Sullivan Park Campus, Corning Incorporated, 21 Lynn Morse Rd, Painted Post, NY 14870, USA

† Ju-Sik Kim and Gabin Yoon contributed equally to this study.

*Ju-Sik Kim: jusik.kim@samsung.com,

*Dongmin Im: dongmin.im@samsung.com

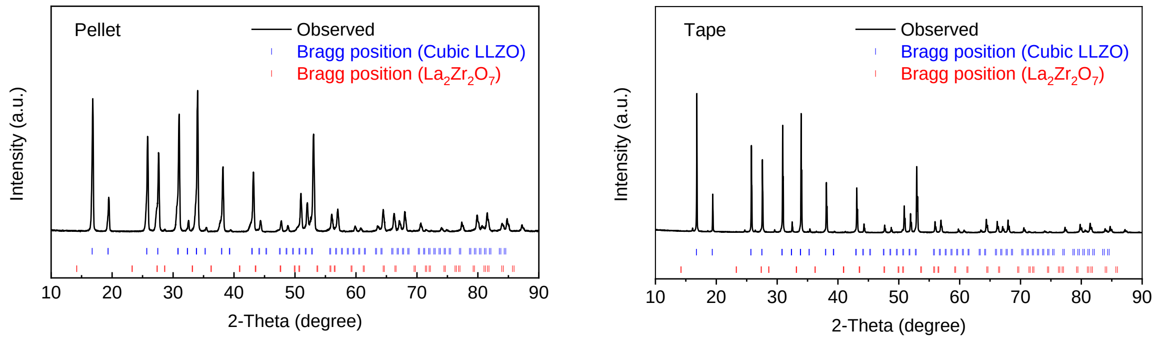

Supplementary Figure 1. **Crystal structure of LLZTO solid electrolytes.** X-ray powder diffraction (XRD) patterns of the pellet and tape LLZTO solid electrolytes.

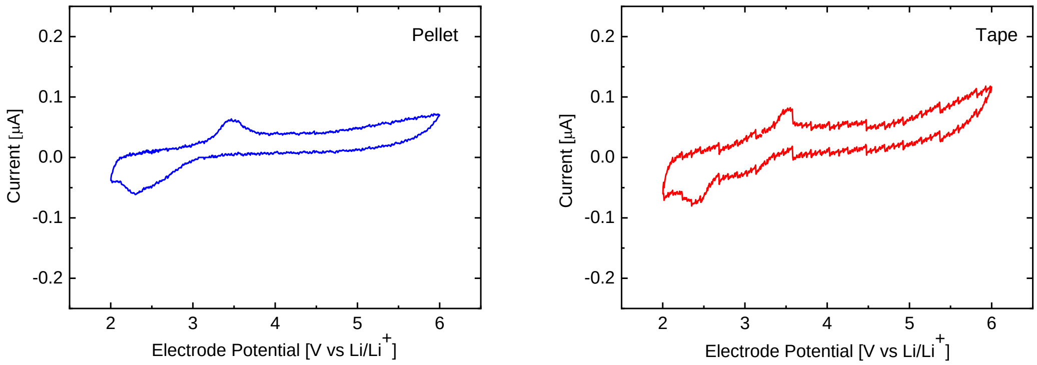

Supplementary Figure 2. **Electrochemical stability of LLZTO solid electrolyte.** Cyclic voltammograms of pellet and tape measured from Au|LLZTO|Li single–layer pouch cells at a scan rate of 0.2 mV s^{-1} at 25 ^{o}C within the voltage range of 2–6 V vs. Li/Li^+.

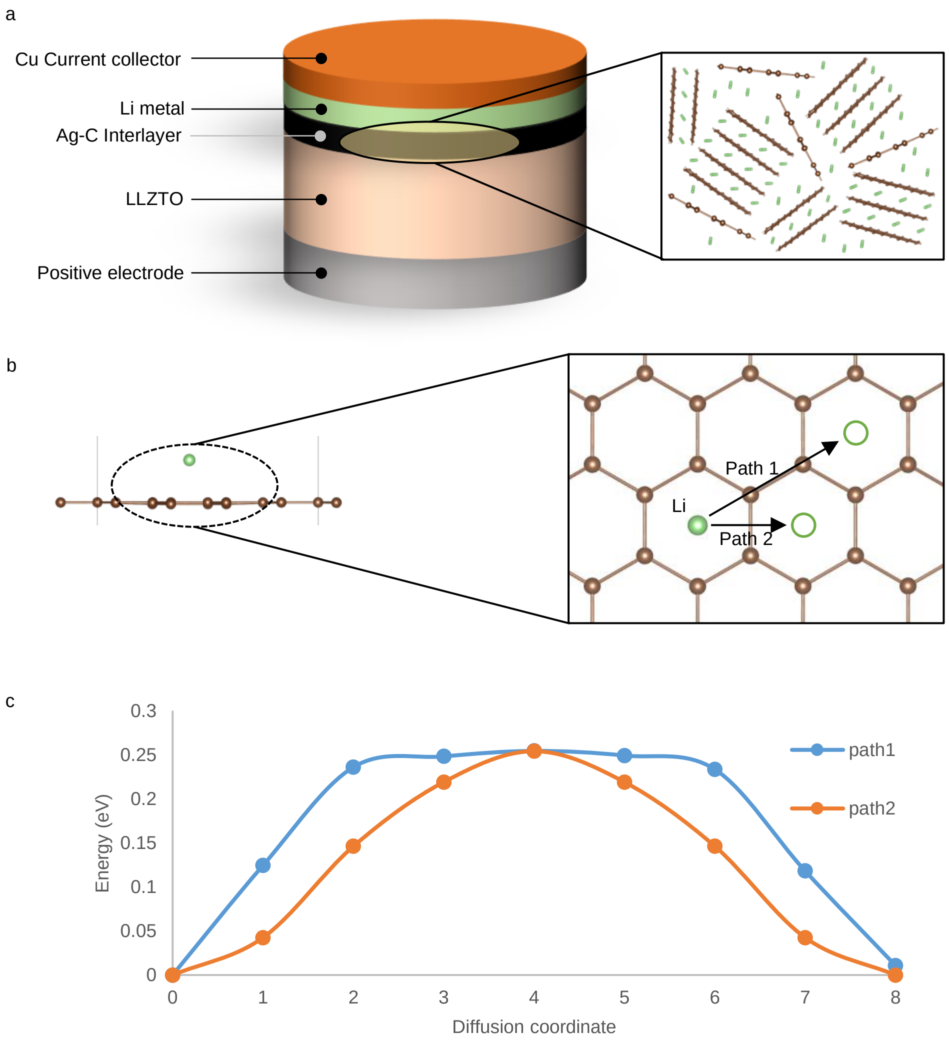

Supplementary Figure 3. **Surface diffusion of Li in the carbon component of Ag-C composite interlayer.** (a) Schematics of cell configuration using LLZTO solid electrolyte and Ag-C interlayer. Enlarged schematic illustrates Li distribution inside Ag-C interlayer. (b) Li diffusion pathways along the surface of carbon component of Ag-C composite interlayer. (c) Energy profiles of Li diffusion along the surface of carbon component of Ag-C composite interlayer.

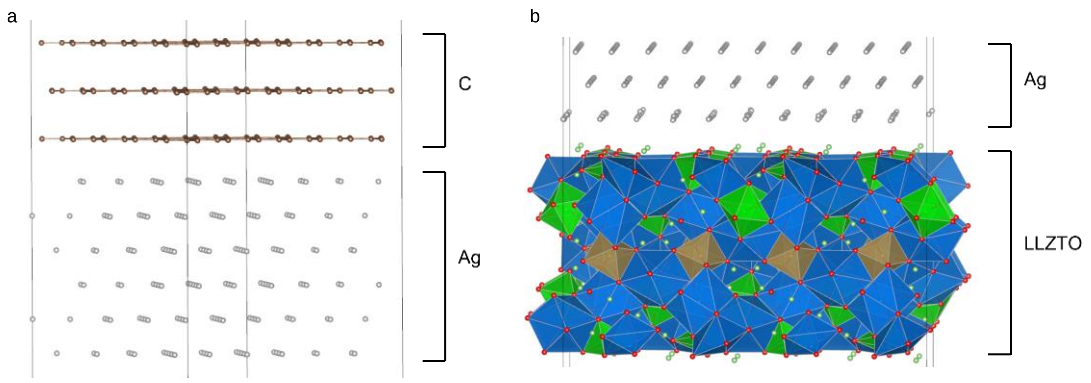

Supplementary Figure 4. **Atomic models of interfaces used for the calculation of adhesion energies.** (a) Ag/C interface model describing Ag/Ag-C interface. (b) Ag/LLZTO interface model.

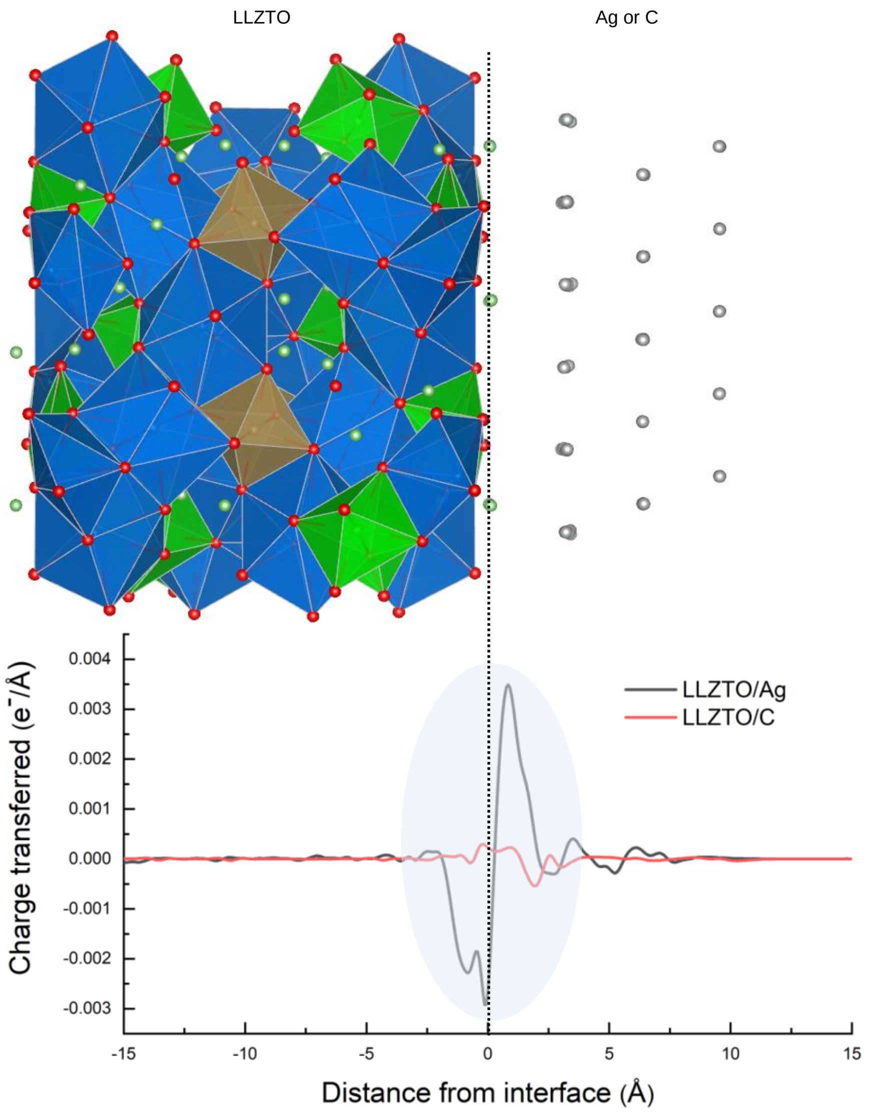

Supplementary Figure 5. **Comparison of plane-averaged differential charge densities at LLZTO/Ag and LLZTO/C interfaces.** It is clearly shown that a larger amount of charge is transferred at LLZTO/Ag interface compared to LLZTO/C interface, which may be responsible for stronger adhesion.

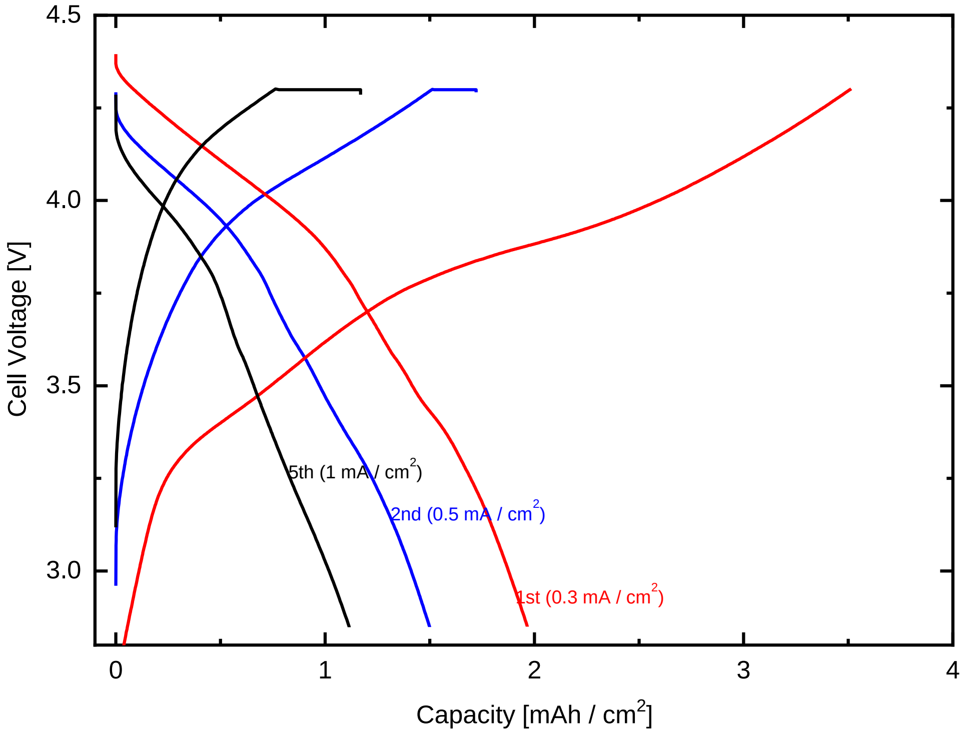

Supplementary Figure 6. **Electrochemical performance of anode-free cell.** Galvanostatic voltage profiles of the Cu|Ag-C/Ag/LLZTO/IL|NCM333 single-layer pouch cell at 25 °C.

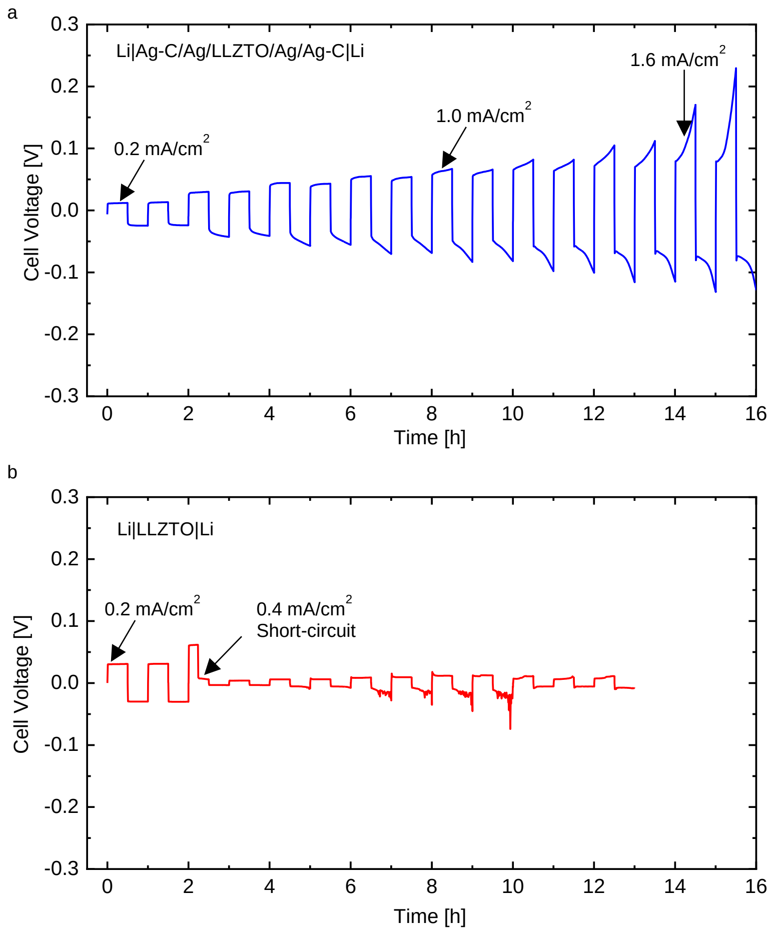

Supplementary Figure 7. **Effect of Ag-C/Ag interlayer on dendrite inhibition.** Galvanostatic cycling of the symmetric pouch cells (a) with Ag/Ag-C interlayer and (b) without interlayer with increasing current densities from 0.2 to 1.6 mA/cm^2 with a step size of 0.2 mA/cm^2 at 25 ^{o}C.

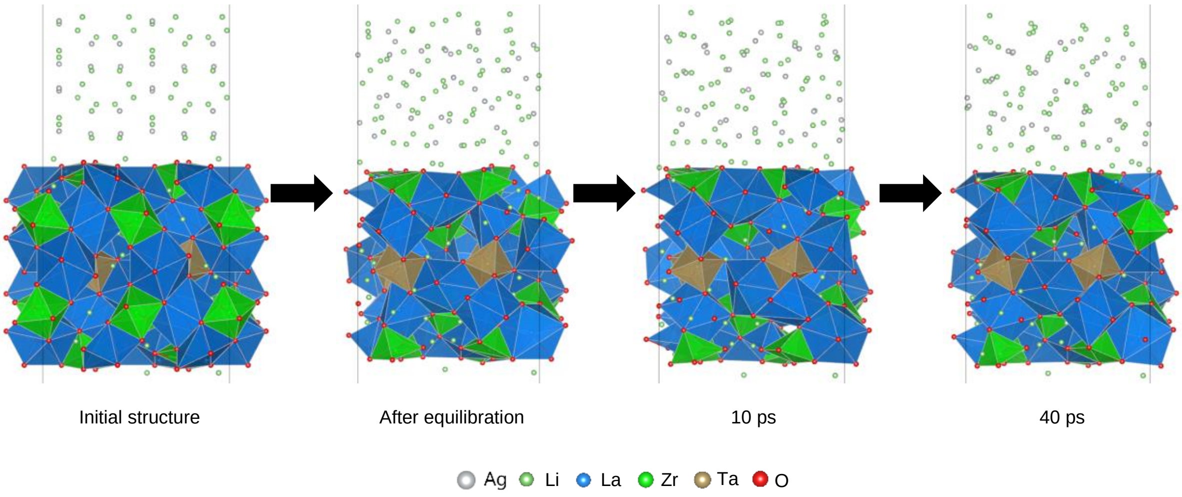

Supplementary Figure 8. **Structure evolution of LLZTO/Li_9Ag_4 interface upon NVT AIMD simulation.** LLZTO/Li_9Ag_4 interface remains intact for at least 40 ps, suggesting that the interface possesses reasonable chemical stability.

Supplementary Table 1. Comparison of conductivities of LLZTO pellet and tape from the symmetric cells (Au | LLZTO | Au) by AC-impedance spectroscopy and Hebb–Wagner polarization method at 25°C. The ionic transference numbers were estimated from the electronic and ionic conductivities: $\boldsymbol{t_{Li^+}} = \frac{\boldsymbol{\sigma_{Li^+}}}{\boldsymbol{\sigma_e} + \boldsymbol{\sigma_{Li^+}}}$. For data reliability, five symmetric cells for each electrolyte were evaluated.

**Sample**	**Ionic conductivity [S/cm]**	**Electronic conductivity [S/cm]**	**Ionic transference number, t_{Li+}**
**LLZTO Pellet**	5.1×10^{-4}	1.46×10^{-9}	0.999997
**LLZTO Tape**	4.4×10^{-4}	2.8×10^{-9}	0.999993

Supplementary Table 2. Calculated adhesion energies of various interfaces. LPSCl stands for argyrodite Li_6PS_5Cl.

**Interface**	LLZTO/C	LLZTO/Li	Cu/C	Cu/Li	Fe/C	Fe/Li	LPSCl/C	LPSCl/Li
**Adhesion energy (J/m^2)**	−0.330	−0.708	−0.595	−1.485	−0.698	−2.051	−0.167	−0.854

Supplementary Table 3. Sums of interfacial adhesion energies in two different Li plating scenarios. Adhesion energy of the Li/interlayer interface was omitted because it is accounted for in both scenarios. Detailed schematics of the two scenarios are shown in Figure 1. SUS stands for stainless steel, and the unit for all numerical values in the table is J/m^2.

**Cases**	**Li plating at the electrolyte/interlayer interface (Figure 1a)**<br><br>Interfaces:<br><br>Collector/Interlayer + Li/Electrolyte	**Li plating at the collector/interlayer interface (Figure 1b)**<br><br>Interfaces:<br><br>Collector/Li + Interlayer/Electrolyte
**Electrolyte: LLZTO**<br>**Current collector: Cu**	(−0.595) + (−0.708) = **(−1.303)**	(−1.485) + (−0.330) = **(−1.815)**
**Electrolyte: LLZTO**<br>**Current collector: Fe (SUS)**	(−0.698) + (−0.708) = **(−1.406)**	(−2.051) + (−0.330) = **(−2.381)**
**Electrolyte: Li_6PS_5Cl**<br>**Current collector: Cu**	(−0.595) + (−0.854) = **(−1.449)**	(−1.485) + (−0.167) = **(−1.652)**

Supplementary Table 4. Fitting parameters determined from complex nonlinear least-squares (CNLS) fitting of the measured impedance spectra in Figure 5b.

**Fitting parameters**	**Anode interlayer**			
	**Ag-C**		**Ag/Ag-C**	
	**Fit values**	**Error (%)**	**Fit values**	**Error (%)**
R_b [Ω cm^2]	11.5	0.20	10.7	0.29
R_1 [Ω cm^2]	39.4	1.05	22.8	1.58
R_2 [Ω cm^2]	27.0	1.57	9.7	4.37
$R_{ct} = R_1 + R_2$ [Ω cm^2]	66.4		32.5	
CPE_1-T	1.17×10^{-6}	1.89	1.29×10^{-6}	2.42
CPE_1-n	0.96	0.39	0.97	0.50
W_R [Ω cm^2]	58.0	0.97	44.4	0.82
W-T	0.21	1.39	0.17	1.16
W-n	0.41	0.35	0.42	0.27
CPE_2-T	2.03×10^{-6}	3.69	5.41×10^{-6}	11.77
CPE_2-n	0.72	0.45	0.70	1.44
chi-square	1.58×10^{-5}		2.32×10^{-5}	

Supplementary Table 5. Parameters considered for estimating energy densities of the full cells at 25 ^{o}C.

**Cathode**	Composition	NCM333
	Areal capacity (mAh/cm^2)	3.2
	Active material (wt%)	96
	Thickness (μm)	50
**Non-aqueous liquid electrolyte additive**	Composition	2M LiFSI in Pyr13FSI
	Amount relative to cathode (wt%)	~ 7
**Solid electrolyte**	Initial thickness (μm)	80
	Thickness after acid treatment	74
**Anode**	Ag/Ag-C interlayer (μm)	6
	Li thickness (μm)	20
**Current collector**	Al for cathode (μm)	9
	Cu for anode (μm)	5
**Total thickness (μm)**		164
**Cell area (cm^2)**		30.2
**Cell volume including package film (L)**		0.540
**Discharge V_{ave} (V)**		3.8
**Energy density (Wh/L)**		680

Supplementary Table 6. Summary of LLZO-based batteries reported in the literature. IL, LE, and PCE stand for ionic liquid, liquid electrolyte, and polymer composite electrolyte, respectively. Full cells, which are reversibly cycled around 100 times at room temperature, are presented.
(*) Thickness of electrolytes are not explicitly reported. Since pellets are used in those studies, we assumed a thickness of 1 mm in accordance with the typical thickness of LLZO pellets.

**Cell structure**	**Cathode capacity (mAh/cm^2)**	**Current density (mA/cm^2)**	**Electrolyte thickness (μm)**	**Number of cycles**	**Reference**
Li|LLZO/LCBO|LCO	0.09	0.006	1000	100	[1]
Li|graphite/LALZWO/IL|NCM523	0.15	0.1	1000	500	[2]
Li|Ge/LLCZNO/Gel|LFP	0.09	0.1	1000 (*)	100	[3]
Li|LLZNO|LCO	0.04	0.004	2000	100	[4]
Li|LLZO/PCE|LFP	0.43	0.04	900	300	[5]
Li|Gel/LLCZNO/Gel|LFP	0.04	0.05	450	70	[6]
Li|LiF-LiCl/LLZTO/IL|LFP	0.79	0.2	1000	100	[7]
Si|LLZTO|LFP	0.04	0.008	1000	100	[8]
Li|LGLZNO/LE|LFP	0.3	0.05	650	50	[9]
Li|LLZTO-Li_3OCl/LE|LFP	0.3	0.05	1000	110	[10]
Li|graphite/LLZTO/LE|LCO	0.18	0.02	130	100	[11]
Li|Sb/LALZO/IL|V_2O_5	0.45	0.05	1000	80	[12]
Li|LLZTO/LE|LFP	0.32	0.07	1000 (*)	100	[13]
Li|Al/LLCZN/LE|LFP	0.13	0.1	300	100	[14]
Li|LE/LLSZSO/LE|LFP	0.45	0.05	1000 (*)	100	[15]
Li|LLZTO/IL|LFP	0.25	0.03	1000	150	[16]
**Li|Ag-C/Ag/LLZTO/IL|NCM**	**2.7**	**1.6**	**80**	**800**	**This work**

References

1. Han, F. *et al.* Interphase Engineering Enabled All-Ceramic Lithium Battery. *Joule* **2**, 497–508 (2018).

2. Shao, Y. *et al.* Drawing a Soft Interface: An Effective Interfacial Modification Strategy for Garnet-Type Solid-State Li Batteries. *ACS Energy Lett.* **3**, 1212–1218 (2018).

3. Luo, W. *et al.* Reducing Interfacial Resistance between Garnet-Structured Solid-State Electrolyte and Li-Metal Anode by a Germanium Layer. *Adv. Mater.* **29**, 1606042 (2017).

4. Ohta, S., Kobayashi, T., Seki, J. & Asaoka, T. Electrochemical performance of an all-solid-state lithium ion battery with garnet-type oxide electrolyte. *J. Power Sources* **202**, 332–335 (2012).

5. Chen, S. *et al.* All-Solid-State Batteries with a Limited Lithium Metal Anode at Room Temperature using a Garnet-Based Electrolyte. *Adv. Mater.* **33**, 2002325 (2021).

6. Liu, B. *et al.* Garnet Solid Electrolyte Protected Li-Metal Batteries. *ACS Appl. Mater. Interfaces* **9**, 18809–18815 (2017).

7. Ruan, Y. *et al.* A 3D Cross-Linking Lithiophilic and Electronically Insulating Interfacial Engineering for Garnet-Type Solid-State Lithium Batteries. *Adv. Funct. Mater.* **31**, 2007815 (2021).

8. Chen, C. *et al.* Sustainable Interfaces between Si Anodes and Garnet Electrolytes for Room-Temperature Solid-State Batteries. *ACS Appl. Mater. Interfaces* **10**, 2185–2190 (2018).

9. Lan, W. *et al.* Realizing Li7La3Zr2O12 garnets with high Li+ conductivity and dense microstructures by Ga/Nb dual substitution for lithium solid-state battery applications. *Sustain. Energy Fuels* **4**, 1812–1821 (2020).

10. Tian, Y. *et al.* Li6.75La3Zr1.75Ta0.25O12@amorphous Li3OCl composite electrolyte for solid state lithium-metal batteries. *Energy Storage Mater.* **14**, 49–57 (2018).

11. Zhang, J., Li, J., Zhai, H., Tan, G. & Tang, X. One-Step Processing of Soft Electrolyte/Metallic Lithium Interface for High-Performance Solid-State Lithium Batteries. *ACS Appl. Energy Mater.* **3**, 6139–6145 (2020).

12. Dubey, R. *et al.* Building a Better Li-Garnet Solid Electrolyte/Metallic Li Interface with Antimony. *Adv. Energy Mater.* **11**, 2102086 (2021).

13. Yang, L. *et al.* Efficient Mutual-Compensating Li-Loss Strategy toward Highly Conductive Garnet Ceramics for Li-Metal Solid-State Batteries. *ACS Appl. Mater. Interfaces* **13**, 56054–56063 (2021).

14. Fu, K. (Kelvin) *et al.* Toward garnet electrolyte–based Li metal batteries: An ultrathin, highly effective, artificial solid-state electrolyte/metallic Li interface. *Sci. Adv.* **3**, e1601659 (2017).

15. Li, X. *et al.* Rational design of strontium antimony co-doped Li7La3Zr2O12 electrolyte membrane for solid-state lithium batteries. *J. Alloys Compd.* **794**, 347–357 (2019).

16. Huo, H. *et al.* In-situ formed Li2CO3-free garnet/Li interface by rapid acid treatment for dendrite-free solid-state batteries. *Nano Energy* **61**, 119–125 (2019).
